# Population Pharmacokinetic Analyses for Plazomicin Using Pooled Data from Phase 1, 2, and 3 Clinical Studies

**DOI:** 10.1128/AAC.02329-18

**Published:** 2019-03-27

**Authors:** Michael Trang, Julie D. Seroogy, Scott A. Van Wart, Sujata M. Bhavnani, Aryun Kim, Jacqueline A. Gibbons, Paul G. Ambrose, Christopher M. Rubino

**Affiliations:** a Institute for Clinical Pharmacodynamics, Inc., Schenectady, New York, USA; b Achaogen, Inc., South San Francisco, California, USA

**Keywords:** aminoglycosides, plazomicin, population pharmacokinetics

## Abstract

Plazomicin is an aminoglycoside with activity against multidrug-resistant *Enterobacteriaceae*. Plazomicin is dosed on a milligram-per-kilogram-of-body-weight basis and administered by a 30-min intravenous infusion every 24 h, with dose adjustments being made for renal impairment and a body weight (BW) of ≥125% of ideal BW. A population pharmacokinetic analysis was performed to identify patient factors that account for variability in pharmacokinetics and to determine if dose adjustments are warranted based on covariates. The analysis included 143 healthy adults and 421 adults with complicated urinary tract infection (cUTI), acute pyelonephritis, bloodstream infection, or hospital-acquired bacterial pneumonia/ventilator-associated bacterial pneumonia (HABP/VABP) from seven studies (phases 1 to 3). A three-compartment structural pharmacokinetic model with a zero-order rate constant for the intravenous infusion and linear first-order elimination kinetics best described the plasma concentration-time profiles. The base structural model included creatinine clearance (CL_CR_) as a time-varying covariate for clearance. The covariates included age, BW, height, body surface area, body mass index, sex, race, and disease-related factors. The ranges of the α-, β-, and γ-phase half-lives for the analysis population were 0.328 to 1.58, 2.77 to 5.38, and 25.8 to 36.5 h, respectively. Total and renal clearances in a typical cUTI or HABP/VABP patient were 4.57 and 4.08 liters/h, respectively. Starting dose adjustments for CL_CR_ are sufficient for minimizing the variation in plasma exposure across patient populations; adjustments based on other covariates are not warranted. The results support initial dosing on a milligram-per-kilogram basis with adjustments for CL_CR_ and BW. Subsequent adjustments based on therapeutic drug management are recommended in certain subsets of patients, including the critically ill and renally impaired.

## Abstract (precis)

Plazomicin is an aminoglycoside with activity against multidrug-resistant *Enterobacteriaceae*. Plazomicin is dosed on a milligram-per-kilogram-of-body-weight basis and administered by a 30-min intravenous infusion every 24 h, with dose adjustments being made for renal impairment and a body weight (BW) of ≥125% of ideal BW.

## INTRODUCTION

Gram-negative bacteria, including common members of the *Enterobacteriaceae* family, have become increasingly resistant to multiple antibiotics over the past decade (1–4). An infection with multidrug-resistant (MDR) pathogens can translate to increased mortality, health care costs, and hospital length of stay (3, 5–8). The limited treatment options currently available for infections due to MDR *Enterobacteriaceae* (9) pose an urgent threat to patients in health care settings; the development of new antibacterial agents is therefore a critical public health priority (1).

Aminoglycosides are an important class of antimicrobials for the treatment of serious bacterial infections due to their proven activity against Gram-negative and Gram-positive pathogens (10–15). Plazomicin is an aminoglycoside that was engineered to overcome aminoglycoside-modifying enzymes, the most common aminoglycoside resistance mechanism in *Enterobacteriaceae* (16). *In vitro*, plazomicin displays rapid bactericidal activity against MDR *Enterobacteriaceae* (17), including isolates that produce extended-spectrum β-lactamases, carbapenemases (18), and/or aminoglycoside-modifying enzymes (19, 20). Plazomicin is approved for the treatment of complicated urinary tract infections (cUTI), including acute pyelonephritis (AP), in patients who have limited or no alternative treatment options (21).

The clinical pharmacokinetic (PK) characteristics of plazomicin are generally consistent with those of other aminoglycoside antibiotics (22). Plazomicin displays linear and dose-proportional PK (23, 24), does not undergo metabolism, and is eliminated from the body primarily via urinary excretion of the parent drug (25). An *in vitro*, equilibrium dialysis experiment that evaluated plazomicin at 5, 50, and 100 μg/ml in human plasma showed that plasma protein binding is concentration independent and low (≈20%; data on file). In a human mass-balance study, 97.5% of the intravenously administered dose of plazomicin was recovered as the parent drug in the urine (25). Given the consistency in PK characteristics with other aminoglycoside antibiotics, plazomicin dosing in patient studies has generally followed typical aminoglycoside dosing recommendations (26, 27), which consider creatinine clearance (CL_CR_) to be a measure of renal function and use adjusted body weight (ABW) when body weight (BW) exceeds the ideal body weight (IBW). Thus, throughout the development program, plazomicin was administered on a milligram-per-kilogram-of-body-weight basis (with ABW in the two phase 3 studies), and dose adjustments were performed in patients based on CL_CR_. The recommended starting dose regimen in the drug product label (21) is plazomicin at 15 mg/kg administered every 24 h (q24h) as a 30-min intravenous infusion in patients with normal renal function or mild renal impairment (CL_CR_ > 60 ml/min).

Given that therapeutic drug management (TDM) is recommended to optimize exposures and clinical outcomes in patients treated with aminoglycosides (28, 29), additional dose adjustments based on TDM were implemented in one of the phase 3 studies (30). In this study, patients with serious infection (bloodstream infection [BSI], hospital-acquired bacterial pneumonia [HABP], ventilator-associated bacterial pneumonia [VABP], cUTI, or AP) due to carbapenem-resistant *Enterobacteriaceae* (CRE) received starting doses based on CL_CR_ and ABW or IBW. Plazomicin doses after the initial dose were determined based on TDM, in which dose adjustments were implemented to maintain the area under the plasma concentration-time curve (AUC) within a target range. AUC-based TDM was considered appropriate for this critically ill patient population; critical illness may be associated with a high PK variability of antibiotics, particularly those that are cleared by the kidneys (31–33).

Although not tested clinically, the plazomicin product label recommends trough concentration-based TDM in patients with cUTI who have a CL_CR_ of >30 to 90 ml/min (i.e., mild to severe renal impairment) to maintain plasma trough concentrations below 3 μg/ml (21). Initial doses of plazomicin are based on CL_CR_ and ABW or IBW in all patients with cUTI, and dose adjustments are necessary in renally impaired cUTI patients. Subsequent doses are informed by the trough concentrations of plazomicin. The rationale for this approach is that cUTI patients with renal impairment may be at increased risk of nephrotoxicity, and trough concentration-based TDM dose adjustments may mitigate this risk (21).

Herein, we describe an analysis that represents the culmination of several population PK modeling activities that were carried out to support the plazomicin clinical development program. The initial population PK model was developed using data from the phase 1 first-in-human study (23), and this model was then more fully elaborated upon with the availability of additional data from phase 1 and 2 studies in healthy subjects and cUTI patients (34–36). Development of the final population PK model, described herein, ensued upon completion of a phase 1 thorough QT study (24) and two phase 3 studies (37, 38). The analysis includes data from patients with cUTI, including AP, and patients with BSI or HABP/VABP due to CRE. The objective of this analysis was to identify patient factors that account for sources of variability in plazomicin PK and to determine if dose adjustments are warranted based on covariates.

## RESULTS

### PK analysis population.

Summary statistics of the baseline characteristics for the analysis population are presented in Table 1. The demographics were diverse and representative of the broader population of adult patients with cUTI/AP and serious infections caused by CRE.

**TABLE 1:** Summary of subject characteristics of the PK analysis population

Variable^*a*^	Values from the following studies:			
	Phase 1 (*n *= 143)	Phase 2 (*n *= 92)	Phase 3 (*n *= 329)	Total (*n *= 564)
Median (range) age (yr)	29 (18–75)	39.9 (18.3–77.4)	64 (18–90)	39 (18–90)
Median (range) BMI (kg/m^2^)	25.9 (18.9–33.5)	25.6 (16.9–38.8)	26.9 (15.4–58.7)	26.0 (15.4–58.7)
Median (range) BSA (m^2^)	1.87 (1.49–2.44)	1.70 (1.32–2.21)	1.89 (1.29–2.58)	1.86 (1.29–2.58)
Median (range) height (cm)	172 (146–191)	160 (142–183)	168 (142–194)	170 (142–194)
Median (range) CL_CR_ (ml/min/1.73 m^2^)	93.4 (7.37–159)	81.3 (21.8–212)	64.6 (8.7–226)	90.2 (7.37–226)
Median (range) body weight (kg)	74.6 (53.5–116)	66.0 (42–100)	76.0 (40.5–165)	75.0 (40.5–165)
No. of subjects with the following characteristics/total no. of subjects (%):				
Male	92/143 (64.3)	15/92 (16.3)	159/329 (48.3)	266/564 (47.2)
Female	51/143 (35.7)	77/92 (83.7)	170/329 (51.7)	298/564 (52.8)
Race				
White	101/143 (70.6)	18/92 (19.6)	324/329 (98.5)	443/564 (78.5)
Black	37/143 (25.9)	13/92 (14.1)	2/329 (0.608)	52/564 (9.22)
Asian	2/143 (1.4)	21/92 (22.8)	1/329 (0.304)	24/564 (4.26)
American Indian/Alaskan Native	1/143 (0.699)	39/92 (42.4)^*b*^	0	40/564 (7.09)
Other	2/143 (1.4)	1/92 (1.09)	2/329 (0.608)	5/564 (0.887)
Infection type				
cUTI	0	40/92 (43.5)	173/329 (52.6)	213/564 (37.8)
AP	0	52/92 (56.5)	112/329 (34.0)	164/564 (29.1)
BSI	0	0	29/329 (8.81)	29/564 (5.14)
HABP/VABP	0	0	15/329 (4.56)	15/564 (2.66)
Healthy	143/143 (100)	0	0	143/564 (25.4)
No. of subjects in the following renal function group/total no. of subjects (%)^*c*^:				
Normal renal function	97/143 (67.8)	39/92 (42.4)	101/329 (30.7)	237/564 (42.0)
Mild renal impairment	32/143 (22.4)	34/92 (37.0)	120/329 (36.5)	186/564 (33.0)
Moderate renal impairment	8/143 (5.59)	19/92 (20.7)	101/329 (30.7)	128/564 (22.7)
Severe renal impairment	6/143 (4.2)	0	7/329 (2.13)	13/564 (2.30)
No. of subjects with the following characteristics/total no. of subjects (%):				
Positive pressure ventilation	0	0	24/329 (7.29)	24/564 (4.26)
Vasopressor use^*d*^	0	0	24/329 (7.29)	24/564 (4.26)
History of trauma^*e*^	0	0	9/329 (2.74)	9/564 (1.60)
Diabetes	0	16/92 (17.4)	47/329 (14.3)	63/564 (11.2)

a The covariate analysis included the following continuous descriptors: age, body weight, height, BSA, and BMI. Evaluated categorical descriptors included sex, race, infection type, positive pressure ventilation, Acute Physiology and Chronic Health Evaluation II (APACHE II) score, history of trauma, vasopressor use, and history of diabetes.

b The phase 2 study case report form recorded geographic ancestry and grouped the following into one category: Americas (Native North American/Native South American/Canadian First Peoples). South American sites were high enrollers and likely account for many of the patients in this category.

c Normal, CL_CR_ ≥ 90 ml/min/1.73 m^2^; mild impairment, CL_CR_ = 60 to 89 ml/min/1.73 m^2^; moderate impairment, CL_CR_ = 30 to 59 ml/min/1.73 m^2^; severe impairment, CL_CR_ < 30 ml/min/1.73 m^2^. In one of the phase 3 studies (study 007), 2/16 patients with mild renal impairment, 3/12 with moderate impairment, and 4/6 with severe impairment received continuous renal replacement therapy at some time during treatment with plazomicin.

d Vasopressor use was defined as administration of adrenaline, dobutamine, etilefrine, isoproterenol, noradrenaline, or norepinephrine at any time during the study. Vasopressors were used as part of the medical management of shock in the critically ill patient population in study 007. Vasopressor use was handled as a categorical covariate (yes/no) to indicate use at any time during the study.

e History of trauma was broadly defined and was commonly unrelated to the clinical condition at the time of plazomicin administration.

### PK data description and outlier analysis.

The final population PK analysis data set included 564 subjects and 4,990 plazomicin plasma concentrations (see Table S1 in the supplemental material), which represented 98.4 and 97.0% of the possible subjects and plasma concentrations available for analysis, respectively. Reasons for sample exclusions included a plazomicin plasma concentration below the lower limit of quantification (BLQ) (46 samples) or designation as an outlier (106 samples).

### Population PK analysis.

**(i) Development of the structural population PK model.** A three-compartment model with zero-order input and first-order elimination best described the pooled plasma plazomicin concentration-time data from the seven studies. Interindividual variability (IIV) was described for total clearance (CL), volume of the central compartment (*V_c_*), distributional clearance to peripheral compartment 1 (CL*_d_*_1_), volume of peripheral compartment 1 (*V_p_*_1_), distributional clearance to peripheral compartment 2 (CL*_d_*_2_), and volume of peripheral compartment 2 (*V_p_*_2_) using log-normal parameter distributions. Residual variability was described using a combined additive-plus-constant coefficient of variation (CCV) error model. CL_CR_ was evaluated as a time-varying covariate for CL in the base structural model, and it was determined that a sigmoidal Hill-type function best described the relationship between CL and CL_CR_. Handling of clearance due to continuous renal replacement therapy (CRRT) is described in Materials and Methods. Given that this model provided an unbiased fit to the data, alternative structural models were not explored further.

All model parameters were estimated with acceptable precision according to the asymptotic errors from the population PK model (standard error of the estimate [SEE], <67%). Goodness-of-fit plots for the base structural population PK model (data not shown) demonstrated that the three-compartment model including the relationship between CL and CL_CR_ provided an excellent fit to the plasma plazomicin concentration-time data across renal function groups. There were no noticeable biases in the model fit when examining these plots, and there was good agreement between both the population mean (coefficient of determination [*r*^2^] = 0.738) and the individual *post hoc* (*r*^2^ = 0.935) predicted concentrations with the observed concentrations.

**(ii) Covariate analysis.** A complete summary of the parameter-covariate relationship determined to be the most statistically significant in each step of forward selection is provided in Table S2. The full multivariable model included 17 parameter-covariate relationships, in addition to the relationship between CL and CL_CR_, which was included *a priori* as part of the structural PK model.

IIV models for the full multivariable model showed that the distributions of each of the individual error terms were normally distributed and symmetric around zero. Revisions were made to the model as part of the full multivariable model assessment. The first step tested a full variance-covariance matrix. Various reduced models estimating different combinations of covariance terms were then evaluated at this stage of the analysis. Parsimony was achieved with the model in which the covariance terms between IIV on clearance (η_CL_) and IIV on volume of the central compartment (η_*V*_*c*__), η_CL_ and IIV on the distributional clearance to peripheral compartment 1 (η_CL_*d*1__), and η_*V*_*c*__ and η_CL_*d*1__ were estimated, resulting in a 364-unit decrease in the minimum value of the objective function (MVOF) (*P < *0.000001). Subsequently, the model with the above-mentioned covariance terms was modified by adding separate error terms for each study phase (phase 1, 2, or 3); this model resulted in a 588-unit drop in the objective function (*P < *0.000001). The final step of model refinement was to test interoccasion variability (IOV) on various model parameters; the occasions were categorized as days 1 to 2, 3 to 6, 6 to 9, and >9. The only IOV term that was found to be statistically significant was the one relating to CL. This refined model was used as the comparator model for backward elimination.

The full multivariable population PK model was then subjected to a backward elimination procedure in which each covariate effect in the model except CL_CR_ was removed in a univariate fashion and tested for statistical significance (α = 0.001). Five rounds of backward elimination were required, and four parameter-covariate relationships (between infection type and *V_p_*_2_ and between age and each of *V_c_*, CL*_d_*_1_, and CL) were removed (Table S2).

**(iii) Final covariate model refinement.** After completing the backward elimination, the model was refined to make it more parsimonious. First, proportional shifts in CL for HABP/VABP and cUTI appeared to have only a minor impact (low fractional change) and were dropped from the model, which resulted in no statistically significant changes (an increase of ≈2 units in the MVOF). Second, proportional shifts in PK parameters based on infection type that had similar estimates (i.e., cUTI and AP, HABP/VABP and BSI) were evaluated as a single group and tested for statistical differences. Finally, the sieving coefficient was reestimated, as it had been fixed to the value from the base structural model (0.926) during covariate analysis; the resultant fitted value was 0.734.

The model that resulted from the steps described above was then subjected to a nonparametric bootstrap procedure. The relationships between *V_p_*_1_ and vasopressor use, CL*_d_*_1_ and HABP/VABP infection type, CL*_d_*_1_ and BSI type, and *V_p_*_1_ and each of HABP/VABP infection and BSI types were dropped from the model due to unacceptably poor precision based on the percent SEE.

**(iv) Final population PK model.** The final population PK model for plazomicin included a fixed zero-order input for the intravenous infusion and first-order elimination. CRRT CL (CL_CRRT_) was estimated only during those periods when CRRT was operative, utilizing the patient-specific and ultrafiltrate flow rate (UFR) and dialysate flow rate (DFR) and the estimated sieving coefficient. IIV was estimated for CL, *V_c_*, CL*_d_*_1_, *V_p_*_1_, CL*_d_*_2_, and *V_p_*_2_ using exponential error models. Random IOV on CL was retained in the model, as it resulted in a statistically significant improvement in the MVOF, despite the small magnitude of the effect (coefficient of variation [CV], 3.59%), which suggests that CL is relatively stable across occasions. An additive-plus-CCV error model best described residual variability with separate CCV error terms for the phase 1 studies, the phase 2 study, and the phase 3 studies.

The population PK parameter estimates and their associated precision (percent SEE) for the fit of the three-compartment model are provided in Table 2. Goodness-of-fit plots (Fig. S1) for the final model showed stronger agreement between the observed plasma concentrations and the population predicted concentrations from the final population PK model (*r*^2^ = 0.793) than from the base structural three-compartment population PK model (*r*^2^ = 0.738). However, the agreement between the observed plasma plazomicin concentrations and the individual *post hoc* predicted concentrations decreased marginally (*r*^2^ = 0.919 versus 0.935 in the base model).

**TABLE 2:** Final population PK model parameter estimates^*a*^

Parameter	Final model		Bootstrap statistics (*n *= 200)	
	Final estimate	% SEE	Mean	90% CI
CL (liters/h)				
Nonrenal CL (liters/h)	0.491	24.9	0.458	0.210 to 0.577
CL_R_ maximum (liters/h)	4.80	7.39	4.86	4.44 to 5.48
Baseline CL_CR50_ (ml/min/1.73 m^2^)	45.3	5.27	45.3	41.9 to 49.8
Hill coefficient	2.49	13.9	2.50	2.01 to 2.93
CL-weight power	0.529	14.0	0.533	0.397 to 0.651
Proportional increase for AP patients	0.130	22.9	0.128	0.0776 to 0.179
Proportional increase for BSI patients	–0.189	41.0	–0.187	–0.300 to 0.0648
*V_c_* (liters)				
Coefficient	9.10	4.07	9.07	8.54 to 9.64
* V_c_*-BSA power	1.23	17.5	1.25	0.869 to 1.59
Proportional increase for cUTI and AP patients	1.05	10.6	1.05	0.867 to 1.23
Proportional increase for BSI and HABP/VABP patients	1.55	17.0	1.56	1.14 to 1.99
CL*_d_*_1_ (liters/h)				
Coefficient	8.05	7.97	8.06	7.09 to 9.15
Proportional increase for cUTI and AP patients	–0.831	4.85	–0.823	–0.880 to 0.748
*V_p_*_1_ (liters)				
Coefficient	8.71	3.97	8.71	8.19 to 9.16
* V_p_*_1_-BSA power	1.17	22.2	1.16	0.670 to 1.72
* V_p_*_1_-age slope	0.00954	11.1	0.00949	0.00796 to 0.0111
Proportional increase for cUTI and AP patients	–0.437	14.6	–0.426	–0.530 to 0.309
CL*_d_*_2_ (liters/h)				
Coefficient	0.199	3.64	0.199	0.186 to 0.215
CL*_d_*_2_-height power	3.38	17.5	3.36	2.15 to 4.43
Proportional increase for cUTI and AP patients	–0.299	46.0	–0.310	–0.533 to 0.0699
Proportional increase for BSI and HABP/VABP patients	2.86	31.7	3.04	1.62 to 5.00
*V_p_*_2_ (liters)				
Coefficient	6.98	9.21	7.00	5.99 to 8.23
* V_p_*_2_-weight power	1.62	20.8	1.53	0.881 to 2.13
Proportional increase for vasopressor use	3.90	36.0	4.15	2.05 to 6.99
CL_CRRT_ (liters/h)				
Sum of UFR and DFR (liters/h)	1.14–1.8	NA	NA	NA
Sieving coefficient	0.734	94.7	0.728	0.405 to 0.999
ω^2^ for CL	0.103	9.52	0.103	0.0870 to 0.120
ω^2^ for *V_c_*	0.211	14.8	0.210	0.156 to 0.270
ω^2^ for CL*_d_*_1_	0.0661	47.8	0.0605	0.00196 to 0.120
ω^2^ for *V_p_*_1_	0.0678	20.9	0.0697	0.0491 to 0.0998
ω^2^ for CL*_d_*_2_	0.0350	24.2	0.0307	0.0165 to 0.0469
ω^2^ for *V_p_*_2_	0.170	34.9	0.128	0.0433 to 0.221
IOV on CL	0.00129	61.1	0.00149	0.000413 to 0.00287
Covariance between CL and *V_c_*	0.0931	15.6	0.0938	0.0701 to 0.122
Covariance between CL and *V_p_*_1_	0.0734	15.1	0.0752	0.0589 to 0.0952
Covariance between *V_c_* and *V_p_*_1_	0.0649	19.6	0.0670	0.0491 to 0.0906
Residual variability, σ^2^				
Additive component	0.0000414	51.5	0.000101	0.0000511 to 0.000179
CCV component for phase 1 studies	0.0297	8.99	0.0299	0.0256 to 0.0345
CCV component for phase 2 studies	0.168	14.9	0.166	0.133 to 0.207
CCV component for phase 3 studies	0.0846	8.78	0.0849	0.0727 to 0.0967

a NA, not applicable; σ^2^, residual variability (sigma squared); ω^2^, interindividual variability (omega squared); CI, confidence interval.

The relationship between renal clearance (CL_R_) and CL_CR_ was described using a sigmoidal Hill-type function, and the relationship between total CL of plazomicin and CL_CR_ included an intercept to represent nonrenal clearance. The other parameters describing the relationship between CL and CL_CR_ were CL_R_ maximum, CL_CR50_, and a Hill coefficient. CL_R_ maximum is the maximum renal clearance (i.e., at a very high value of CL_CR_), CL_CR50_ is the CL_CR_ value at which CL_R_ is half-maximal, and the Hill coefficient defines the shape of the sigmoidal relationship. Based upon these relationships, the population mean total CL and CL_R_ would be 4.57 liters/h (76.2 ml/min) and 4.08 liters/h (68.0 ml/min), respectively, in a typical cUTI or HABP/VABP patient with a BW of 75 kg, a body surface area (BSA) of 1.73 m^2^, and a CL_CR_ of 90 ml/min.

As summarized in Table 2, several statistically significant relationships were identified between covariates and IIV for PK parameters: BW and infection type on CL; BSA and infection type on *V_c_*; infection type on CL*_d_*_1_; BSA, age, and infection type on *V_p_*_1_; height and infection type on CL*_d_*_2_; and BW and vasopressor use on *V_p_*_2_.

### Model evaluation.

**FIG 1:**
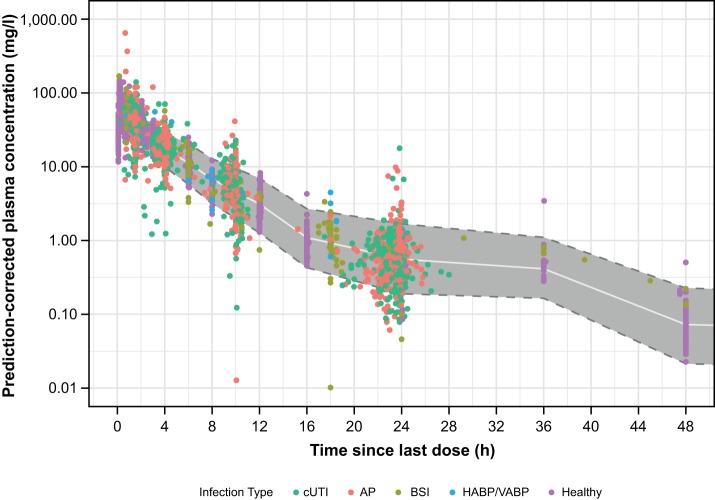
Prediction-corrected visual predictive check for the final population PK model for plazomicin in healthy subjects and patients over the first 48 h after a dose, stratified by infection type. Dashed lines with gray zone, 5th and 95th percentiles of simulated concentrations; white line, median or 50th percentile of simulated concentrations; dots, observed plasma concentrations.

The prediction-corrected visual predictive check (PC-VPC) plots generally showed reasonable agreement between the observed concentrations and the individual simulated concentrations across time intervals and showed no bias with respect to renal function, infection type, or first versus multiple doses across healthy subjects and patients (Fig. 1), which suggests no substantial issues with respect to the fixed or random-effects parameters in the model and which supports the future use of this model for simulations. Furthermore, the bootstrap analysis showed that all of the population PK model parameters were estimated with reasonable precision (Table 2).

### Plazomicin exposures and secondary PK parameter estimates.

Summary statistics for the key exposures and secondary PK parameters are provided in Table 3. The plazomicin half-lives (*t*_1/2_) associated with the three-compartment structural model indicate an initial distribution phase (α-phase half-life, 0.328 to 1.58 h), followed by a secondary distribution phase (β-phase half-life, 2.77 to 5.38 h) and a terminal elimination phase (γ-phase half-life, 25.8 to 36.5 h).

**TABLE 3:** Summary of key plazomicin PK parameters in healthy subjects and patients, derived from the population PK model

Parameter	Geometric mean value (% CV)^*d*^			
	Study 006	Study 002	Study 009	Study 007
AUC_0–24_ (mg·h/liter)^*a*^	248 (16.0)	233 (43.4)	234 (38.5)	235 (42.0)
CL (liters/h)	4.50 (14.3)	4.43 (41.3)	3.91 (42.5)	2.91 (60.5)
*C*_max_ (mg/liter)^*b*^	84.6 (21.0)	54.5 (41.4)	46.6 (43.0)	37.1 (39.3)
*C*_min_ (mg/liter)^*c*^	0.372 (45.9)	0.494 (104)	0.880 (95.4)	2.10 (99.4)
*t*_1/2,α_ (h)	0.328 (27.2)	1.30 (26.7)	1.58 (31.2)	0.623 (45.7)
*t*_1/2,β_ (h)	2.77 (17.3)	3.95 (26.2)	5.17 (31.6)	5.38 (46.9)
*t*_1/2,γ_ (h)	25.8 (37.6)	36.5 (32.2)	36.1 (31.3)	28.6 (86.9)
*V*_ss_ (liters)	25.0 (23.9)	27.9 (30.4)	31.5 (32.9)	56.6 (49.7)

a For phase 2/3 studies, AUC_0–24_ is the average daily AUC calculated via numerical integration of the concentration-time profile from the time of the first dose until 48 h divided by 2. For study 006, AUC_0–24_ is the AUC for first the 24 h after the single 15-mg/kg dose.

b For phase 2/3 studies, *C*_max_ is the highest concentration observed in the first 48 h of therapy (typically, the end of infusion after the second dose for patients receiving q24h dosing and the end of infusion after the first dose for those receiving q48h dosing). For study 006, *C*_max_ is the highest concentration in the first 24 h after the single 15-mg/kg dose (always at the end of infusion).

c For phase 2/3 studies, *C*_min_ is the lowest concentration observed in the first 48 h of therapy (typically, the trough concentration after the first dose). For study 006, *C*_min_ is the lowest concentration in the first 24 h after the single 15-mg/kg dose (always at 24 h).

d In study 006, plazomicin was administered at 15 mg/kg to 54 healthy subjects. In study 002, plazomicin was administered at 15 mg/kg to 71 cUTI/AP patients. In study 009, the dose was based on the baseline renal function, as described in Table 4, footnote *a*, and was administered to 281 cUTI/AP patients. In study 007, the dose was based on the baseline renal function, as described in Table 4, footnote *a*, and was administered to 48 CRE patients. Two subjects in study 007 received q12h dosing starting at ≈24 h after the first dose of plazomicin, subsequent to therapeutic drug management.

Table 3 reports PK results for 54 healthy subjects and 71 patients with cUTI/AP who received plazomicin at 15 mg/kg by a 30-min infusion. The mean peak plasma concentration (*C*_max_) values were 84.6 mg/liter (CV, 21.0%) and 54.5 mg/liter (CV, 41.4%), respectively, indicating that the 15-mg/kg dose produced a higher *C*_max_ in healthy subjects than in patients with cUTI/AP. This is likely attributed to a smaller *V_c_* in healthy subjects than in patients with cUTI/AP. Overall, the mean volume of distribution at steady state (*V*_ss_) of plazomicin in healthy subjects and patients with cUTI/AP ranged from 25.0 to 31.5 liters, which is typical for an aminoglycoside and approximately twice the extracellular fluid volume (39). A larger *V*_ss_ of 56.6 liters was observed in BSI and HABP/VABP patients with CRE infections, most likely due to the severity of illness in this patient group (40). The estimates of the mean plazomicin area under the plasma concentration-time curve from time zero to 24 h (AUC_0–24_) for a plazomicin dose of 15 mg/kg or a reduced dose based on the baseline renal function were generally consistent across the patient studies with moderate variability (38.5 to 43.4%). Mean plazomicin minimum plasma concentration (*C*_min_) values ranged from 0.494 to 0.880 mg/liter for patients with cUTI/AP and were higher (2.10 mg/liter) in patients with CRE infections.

To aid in identifying subgroups of patients who are likely to experience important differences in plazomicin exposures, the *post hoc* plazomicin PK estimates (AUC_0–24_, *C*_max_, *C*_min_, and CL) were investigated relative to patient covariates of interest (CL_CR_, BW, body mass index [BMI], age, sex, race, infection type, and vasopressor use). The assessments of clinical relevance focused on AUC and *C*_min_ in patients in the phase 2 and phase 3 studies. AUC is of interest for plazomicin because the AUC/MIC ratio is the PK/pharmacodynamic (PD) index that best correlated with efficacy in an animal infection model (41). *C*_min_ is of interest based on the precedent of clinical use with other aminoglycoside antibiotics, which commonly involve monitoring of *C*_min_ to improve the safety profile. While the exposure results reported below are confined to AUC, the same conclusions were reached for exposure based on *C*_min_.

**FIG 2:**
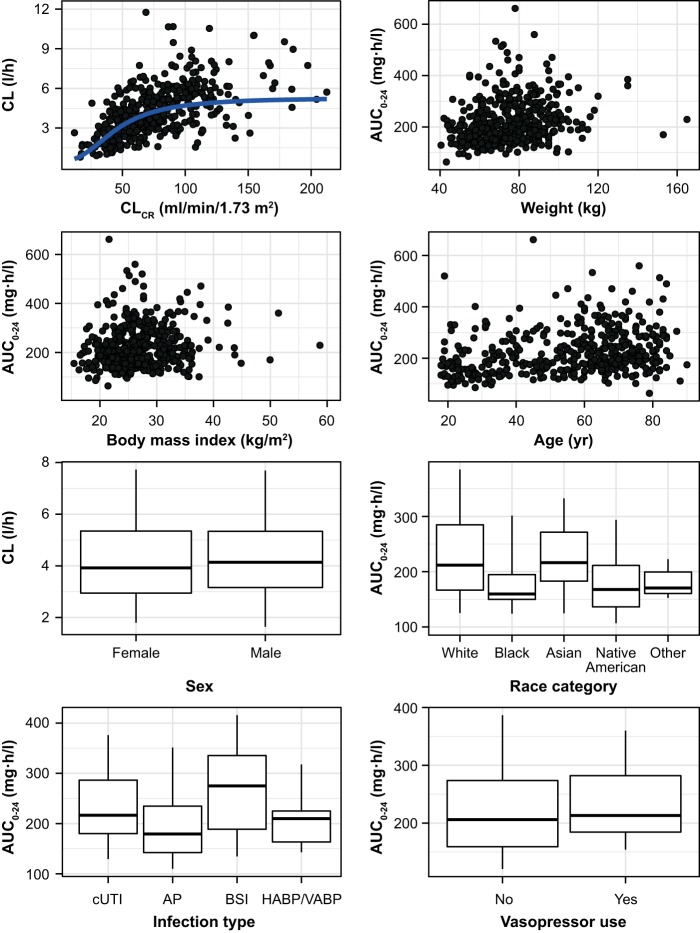
Relationships between *post hoc* plazomicin parameter estimates (AUC or CL) following initial dosing and covariate category (CL_CR_, BW, BMI, age, sex, race, infection type, or vasopressor use) for patients in the phase 2 and 3 studies. The horizontal lines in box-whiskers plots are the median; the boxes show the 25th to 75th percentiles, and whiskers extend to the 5th and 95th percentiles.

Figure 2 presents the relationships between *post hoc* parameter estimates for AUC_0–24_ and CL and the covariates. Total CL increased in a sigmoidal fashion with increasing CL_CR_. Given that the initial dose was selected based on the baseline CL_CR_ in the phase 3 studies, AUC_0–24_ was similar across renal function groups. The scatter plots show no important trends between AUC_0–24_ and each of BW, BMI, or age. Box plots show broadly overlapping values of CL in males and females, as well as broadly overlapping values of AUC_0–24_ in patients who received vasopressors versus those who did not. Box plots of AUC_0–24_ versus race suggest that black patients may have slightly lower plasma exposures than patients of other races; however, race was not a significant covariate in the final population PK model, and apparent differences among racial categories are likely due to small sample sizes and potential imbalances in other factors (e.g., renal function, infection type). Box plots of AUC_0–24_ versus infection type suggest slightly higher plasma exposures in patients with BSI, which reflects an 18.9% decrease in CL relative to that in patients with cUTI or HABP/VABP (Table 2).

## DISCUSSION

The objective of this analysis was to identify patient factors that account for sources of variability in plazomicin PK and to determine if dose adjustments are warranted based on covariates. The population PK model for plazomicin that best described the data was a three-compartment model with a zero-order rate constant for the intravenous infusion and with first-order elimination kinetics. The relationship between CL_R_ and CL_CR_, the most clinically significant covariate, was described by a sigmoidal Hill-type function. As glomerular filtration is the predominant mechanism of aminoglycoside elimination, the relationship between plazomicin CL and the term used to capture renal clearance might be expected to be linear. However, the observed data showed a sigmoidal relationship, where increases in plazomicin CL were less pronounced at the highest CL_CR_ values (Fig. 2). It is important to consider that CL_CR_ is used in this empirical population PK model as a surrogate of glomerular filtration rate (GFR), and CL_CR_ may be less predictive of GFR at the upper range of CL_CR_ estimates due to other factors which influence endogenous creatinine production. Despite this limitation, the sigmoidal relationship supports the objective of this analysis (i.e., identifying important patient covariates for plazomicin CL). It should also be noted that data were sparse for CL_CR_ of >150 ml/min; therefore, caution should be exercised when using the model to predict exposures in subjects with CL_CR_ above this level.

Plazomicin dosing was based on adjustments for the baseline CL_CR_ in the phase 3 clinical studies (studies 007 and 009; Table 3), and these dosage adjustments are reflected in the product label (21). As the clearance of plazomicin is primarily through glomerular filtration, it may be reasoned that plazomicin dosing should be based on GFR. The gold standard for estimating GFR is by using inulin, which is highly precise but impractical for routine use in guiding drug dosing (42). The Cockcroft-Gault (CG) formula estimates CL_CR_ as a surrogate for GFR, and most guidelines for aminoglycoside dosing recommend use of the CG formula. This formula requires the patient weight, which may be adjusted for IBW, lean BW, or BSA to account for differences in body size and obesity among patients (43). Alternative equations for estimated GFR (eGFR) are available, such as the Modification of Diet in Renal Disease (MDRD) and Chronic Kidney Disease Epidemiology Collaboration (CKD-EPI) formulas (44, 45). These eGFR formulas were developed from well-conducted population studies and are primarily used to detect end-stage chronic kidney disease. While they represent an alternative to the CG formula to guide aminoglycoside dosing, they have not been as widely adopted as the CG formula. Moreover, studies suggest that the CG, MDRD, and CKD-EPI formulas are not interchangeable and may result in different renal function estimates, leading to differences in antimicrobial dosing. Given these caveats, the population PK analysis of plazomicin focused on CG, which was used in the phase 3 clinical studies (Table 4) and was thus integral to the overall assessment of benefit-risk in the intended patient populations.

**TABLE 4:** Overview of the clinical studies included in the analysis^*c*^

Study phase	Study no. (reference)	Plazomicin PK population	Study design	Plazomicin dosing^*a*^	PK sampling regimen type and times
Phase 1	001 (23)	Healthy subjects (*n *= 28)	Randomized, double blind, placebo controlled, parallel group	1–15-mg/kg single dose; 4–15 mg/kg q24h	Intensive (predose and at 5, 10, 15, 20, 30, and 45 min and 1, 2, 3, 4, 6, 8, 12, 16, 24, and 48 h after the start of the infusion)
	003 (34)	Healthy subjects (*n *= 30)	Randomized, double blind, placebo controlled	10.7- or 15-mg/kg single dose; 15 mg/kg q24h	Intensive (predose and at 10, 15, 30, and 45 min, and 1, 1.5, 2, 3, 6, and 10 h or 1, 1.5, 2, 2.5, 3, 4, 6, 8, 12, 16, and 24 h after the start of the infusion)
	004 (36)	Healthy subjects with normal renal function or with various degrees of renal impairment (*n *= 24)	Open label	7.5-mg/kg single dose	Intensive (predose and at 36 and 45 min and 1, 1.5, 3, 6, 10, 16, 24, 36, 48, 72, and 96 h after the start of the infusion)
	006 (24)	Healthy subjects (*n *= 61)	Randomized, double blind, placebo and positive controlled, crossover	15- or 20-mg/kg single dose	Intensive (predose and at 36 and 45 min and 1, 2, 3, 6, 12, and 24 h after the start of the infusion)
Phase 2	002 (35)	Patients with cUTI or AP (*n *= 92)	Double blind, comparator controlled	10 or 15 mg/kg q24h	Sparse (at 35–55 min and 1.5–3 h after the start of the infusion on day 1 and just before the start of the infusion on days 2–5)
Phase 3	007 (38)	Patients with BSI, HABP, VABP, or cUTI due to CRE (*n *= 48)	Randomized, open label, compared with colistin	Initial dose of 8–15 mg/kg q12h, q24h, or q48h based on baseline renal function,^*b*^ followed by doses based on TDM	At 1.5, 4, and 8, 10, or 18 h after the start of infusion on days 1 and 4 or at 0.75 and time points up to 12 (q12h), 24 (q24h), or 48 (q48h) h after the start of infusion on day 1 and at the end of therapy or early discontinuation
	009 (37)	Patients with cUTI, including AP (*n *= 281)	Randomized, double blind, compared with meropenem	15 mg/kg q24h with dosing adjustments to 8–15 mg/kg q24h per renal function^*b*^	Predose, 90 min, 4 h, and 10 h after the start of infusion relative to the first dose of study drug on the day of sampling

a In all studies except for studies 007 and 009, milligram-per-kilogram dosing was based strictly on total BW. In studies 007 and 009, milligram-per-kilogram dosing was based on BW except where BW was ≥125% of the ideal body weight (IBW). IBW was calculated based on sex and height, as described by Devine (53). Where BW was ≥125% of IBW, patients in studies 007 and 009 were dosed based on ABW, which was calculated as follows: ABW = IBW · 0.4 (BW – IBW) (27).

b Renal function was determined by use of the calculated CL_CR_, as estimated by the equation of Cockcroft and Gault (46), with the use of ABW where BW was ≥125% of IBW, as described in footnote *a*. In studies 007 and 009, the dose schedules and the CL_CR_ categories were as follows: 15 mg/kg q24h for >60 ml/min, 12 mg/kg q24h for >50 to 60 ml/min, 10 mg/kg q24h for >40 to 50 ml/min, 10 mg/kg q24h for >40 to 50 ml/min, and 8 mg/kg q24h for >30 to 40 ml/min. Study 007 included the following: 12 mg/kg q48h for >25 to 30 ml/min; 10 mg/kg q48h for >20 to 25 ml/min; 8 mg/kg q48h for >15 to 20 ml/min; 8 mg/kg q48h for ≤15 ml/min; 10 mg/kg q12h for CRRT, slow; and 11 mg/kg q24h for CRRT, fast. CRRT, slow, assumed residual plazomicin renal clearance of 0 ml/min and clearance attributed to a CRRT of 2.4 liters/h using slow dialysate and ultrafiltrate flow rates (5 to 15 ml/min) and a blood flow rate of 150 ml/min. CRRT, fast, assumed residual plazomicin renal clearance of 0 ml/min and clearance attributed to CRRT of 4.8 liters/h using fast dialysate and ultrafiltrate flow rates (30 to 40 ml/min) and a blood flow rate of 150 ml/min.

c q12h, once every 12 h; q24h, once every 24 h; q48h, once every 48 h.

The population PK analysis used traditional covariate model-building techniques to identify patient descriptors that are associated with the IIV in plazomicin PK. After pooling of the data across phase 1, 2, and 3 studies, the resultant data set contained a large, diverse population, which facilitated the identification of several statistically significant relationships (Table 2). Infection type appeared to reduce IIV in a variety of PK parameters, especially CL*_d_*_1_, which may reflect changes in hemodynamic status and other critically ill patient characteristics with the inclusion of patient data from the phase 3 study in patients with serious infections due to CRE. The relatively small drop in IIV for CL is a consequence of the fact that CL_CR_, which was part of the base structural model, is by far the largest predictor of the IIV in plazomicin CL.

*Post hoc* assessments, which showed a compelling relationship between CL and CL_CR_, provided support for dose adjustments based on CL_CR_. In contrast, none of the other statistically significant covariates had a clinically meaningful impact on plazomicin exposure. Thus, dose adjustments in adults do not appear to be warranted on the basis of age, infection type, vasopressor use, or body size (further to milligram-per-kilogram dosing using either total BW or ABW).

*Post hoc* assessments further enabled comparison of AUC_0–24_ values in the two phase 3 studies (studies 007 and 009; Table 3). The geometric mean AUC_0–24_ was 235 mg·h/liter (CV, 42.0%) in patients with infections due to CRE in study 007 and 234 mg·h/liter (CV, 38.5%) in patients with cUTI/AP in study 009, indicating that the dosing paradigm produced only moderate variability in the exposure parameter that is considered most relevant to efficacy. This suggests that it is appropriate for plazomicin to be administered on a milligram-per-kilogram basis with adjustments for CL_CR_ and BW of ≥125% of IBW.

In conclusion, a three-compartment structural PK model with a zero-order rate constant for the intravenous infusion and linear first-order elimination kinetics provided good fits of the plasma concentration-time data. A robust description of the plasma PK of plazomicin in the population of healthy subjects and patients was achieved, such that the derived measures of plazomicin exposure are expected to be both accurate and precise. The population PK analysis successfully identified patient factors that are sources of variability in PK parameters, while further showing that most of these covariate relationships do not have an important impact on plasma exposure to plazomicin. *Post hoc* estimates of AUC_0–24_ in the phase 3 studies suggest that it is appropriate for plazomicin to be administered on a milligram-per-kilogram basis with dose adjustments for CL_CR_ and BW of ≥125% of IBW, while adjustments based on other covariates are not warranted. Although it was beyond the scope of the population PK analysis, it merits mention that initial dosing on a milligram-per-kilogram basis with adjustments for CL_CR_ and BW of ≥125% of IBW is recommended in all patients, and subsequent adjustments based on TDM are recommended in certain patient subsets, including the critically ill and those with cUTI/AP and coexisting renal impairment.

## MATERIALS AND METHODS

### Study designs.

The clinical studies involved in this analysis included four phase 1 studies (studies 001 [ClinicalTrials.gov registration no. NCT00822978] [23], 003 [ClinicalTrials.gov registration no. NCT01034774] [34], 004 [ClinicalTrials.gov registration no. NCT01462136] [36], and 006 [ClinicalTrials.gov registration no. NCT01514929] [24]), one phase 2 study (study 002 [ClinicalTrials.gov registration no. NCT01096849] [35]), and two phase 3 studies (studies 007 [ClinicalTrials.gov registration no. NCT01970371] [38] and 009 [ClinicalTrials.gov registration no. NCT02486627] [37]).

Table 4 summarizes the clinical studies and their dosing and PK sampling schemes. The phase 1 studies were conducted in healthy subjects in whom plazomicin was administered strictly on a milligram-per-kilogram basis (no adjustments for CL_CR_ or IBW). Studies 001 and 003 explored the safety, tolerability, and PK of plazomicin over single- and multiple-dose regimens. Study 004 explored the effect of renal impairment on the PK and tolerability of single-dose plazomicin. Study 006 assessed the potential of single-dose plazomicin to cause QT prolongation.

Three multiple-dose studies were performed in patients with cUTI, including AP (studies 002 and 009) or serious infection (BSI, HABP, VABP, cUTI, or AP), due to CRE (study 007). In all three patient studies, plazomicin blood samples were collected using sparse PK sampling schemes (Table 4). Studies 002 and 009 were randomized phase 2 and phase 3 cUTI trials, respectively, where plazomicin was compared with levofloxacin or meropenem, respectively. Study 007 was a randomized phase 3 trial of plazomicin versus colistin in critically ill patients with infections due to CRE. In study 002, plazomicin was administered at 10 or 15 mg/kg based strictly on BW, and in studies 007 and 009, plazomicin was administered at up to 15 mg/kg with adjustment for renal function and ABW where BW was ≥125% of IBW (see Table 4 footnotes). In study 007, plazomicin doses after the initial dose were determined based on therapeutic drug management, in which dose adjustments were implemented to maintain AUC within a target range.

### Subject characteristics.

Subject demographic and disease characteristics recorded before administration of study drug were used to characterize the analysis population and informed assessments of IIV in key PK parameters.

Renal function was approximated using CL_CR_, as calculated by the CG equation (46) as follows: CL_CR_ (in milliliters per minute) for males = (140 – age [in years] · BW [in kilograms])/(72 · SCr [in milligrams per deciliter]) and CL_CR_ for females (in milliliters per minute) = male CL_CR_ · 0.85, where the serum creatinine concentration (SCr) was capped to a lower bound of 0.50 mg/dl. The calculated CL_CR_ values for each individual were normalized to a BSA of 1.73 m^2^, determined using the equation from Du Bois and Du Bois (47); BSA (in millimeters squared) = BW (in kilograms)^0.425^ · height (in centimeters)^0.725^ · 0.007184, to help control for body size differences when estimating renal function for the purposes of constructing a population PK covariate model.

If SCr data were available on different days during repeated dosing of plazomicin within a given patient, CL_CR_ was calculated and updated for each day where SCr was measured and used as a time-changing covariate in the analysis data set. Before updating the CL_CR_ calculation, linear interpolation was used to calculate SCr between actual measured SCr values.

For patients on CRRT, the timing of CRRT, UFR, and DFR was assigned based upon the source data.

### Drug concentration assay.

In each study, blood samples for PK analysis were collected in Vacutainer tubes containing K_2_EDTA. Plasma was separated from whole-blood components by centrifugation and immediately frozen at –20°C or colder until analysis (Alturas Analytics, Inc. Moscow, ID, USA). Plasma plazomicin concentrations were determined using a validated liquid chromatography-tandem mass spectrometry method with a lower limit of quantification of 0.01 mg/liter. The same assay methodology was used for every study included in the population PK analysis.

### Handling of outliers and samples assayed as having BLQ plazomicin plasma concentrations.

PK samples without both date and time information or with BLQ plazomicin concentrations were excluded from the population PK analysis.

An outlier was defined as an aberrant observation that substantially deviated from the rest of the observations within an individual. PK outlier concentrations were excluded from this analysis according to U.S. Food and Drug Administration guidance (48). The outlier detection was based primarily upon visual inspection of individual and pooled plasma concentration-time data for plazomicin. Searching for additional outliers during the analysis was based upon graphical exploration of individual and population conditional weighted residuals during structural PK model development.

### Population PK analysis.

The population PK analysis was conducted using NONMEM software (v7.2; ICON Development Solutions, Ellicott City, MD, USA), implementing the first-order conditional estimation method with interaction. During various stages of model development, population PK models were minimally assessed using the following criteria: (i) evaluation of individual and population mean PK parameter estimates for plazomicin and their precision, as measured by the percent standard error of the population mean estimate; (ii) graphical examination of standard diagnostic and population analysis goodness-of-fit plots with possible stratification by various factors, such as patient population or plazomicin dose group; (iii) graphical examination of the agreement between the observed and individual *post hoc* predicted plazomicin concentration-time data; (iv) reductions in both residual variability and IIV; and (v) comparison of MVOF for nested models or Akaike’s information criterion for nonnested models (49).

**(i) Development of the structural population PK model.** For developing the structural population PK model, a previously developed three-compartment structural PK model using data from the phase 1 and 2 studies (studies 001, 003, 004, and 002), in which CL, *V_c_*, *V_p_*_1_ and *V_p_*_2_, and CL*_d_*_1_ and CL*_d_*_2_ were estimated as model parameters, was refined in this analysis after including additional data from studies 006, 007, and 009 pooled with the previous phase 1 and 2 study data. As a general rule, other model structures (e.g., a two-compartment model) were attempted only if it was deemed necessary based upon the fit of the structural model to the pooled data set. Development of the base structural population PK model for the present analysis included CL_CR_ as a time-varying covariate *a priori* for all subjects in the full data set who had more than one central laboratory SCr measurement. The functional form for the relationship between CL_CR_ and CL was selected based upon the fit of different functional forms (i.e., linear, power, sigmoidal). Clearance due to CRRT was set to the sum of the actual patient-specific DFR and UFR, and multiplied by an estimate of the sieving coefficient (which represents the membrane permeation of the drug) on the study days when CRRT was used. For this submodel, DFR and UFR were fixed based on the source data and the sieving coefficient was a fitted parameter. The total CL for patients while on CRRT was equal to the sum of residual CL and the CRRT CL.

IIV was modeled for each PK parameter, where appropriate, using an exponential error model that assumed that these parameters are log-normally distributed and that the variance is constant. Residual variability was initially modeled by a combined additive-plus-CCV error model. Other models for residual variability were explored as necessary.

**(ii) Covariate analysis.** After constructing the structural population PK model, a covariate analysis was initiated. Patient factors were evaluated as continuous or categorical descriptors, as summarized in Table 1.

A formal univariate analysis of each covariate that demonstrated an observable trend with a structural PK model parameter and that had a biologically plausible relationship was performed in NONMEM during each step of forward selection. This was carried out to assess statistical significance based upon the resulting decrease in MVOF from the base structural model using a likelihood ratio test. The most statistically significant parameter-covariate relationship (α = 0.01) was added to the model during each step of forward selection; stepwise forward selection was concluded when none of the remaining covariates tested resulted in a statistically significant decrease in MVOF relative to that for the updated base structural model from the previous step of forward selection.

After completing forward selection, the refinement of the full multivariable model was conducted. The IIV models were first reevaluated through pairwise comparisons of the interindividual error terms (η) for each parameter, and potential adjustments to the IIV models or the variance-covariance matrix structure were made. Focus was then shifted toward correcting any potential biases or seeking ways to simplify the residual variability model, and if necessary, the additive-plus-CCV residual error model was simplified to a CCV error model at this stage of the analysis.

Univariate stepwise backward elimination was performed after all adjustments were made to the IIV and residual variability models by removing each parameter-covariate relationship and assessing whether the resulting increase in MVOF remained statistically significant (α = 0.001). The final population PK model for plazomicin was generated after it was determined that all parameter-covariate relationships in the model remained statistically significant.

**(iii) Final model evaluation.** The final population PK model analysis was qualified by performing a PC-VPC, which examined the agreement between the 5th, 50th, and 95th percentiles of the observed and the individual simulated plazomicin concentrations across time intervals. The PC-VPC normalizes both the observed and the simulated plasma concentration-time data by the median population mean predictions during each time interval to adjust for the differences due to independent variables in the final population PK model and to avoid having to stratify by single- versus multiple-dose data, dose group, or other significant covariate effects included in the model (50). This was also necessary due to the fact that patients could have doses altered secondary to TDM in study 007.

To assess the robustness of the final population PK model for plazomicin, a nonparametric bootstrap evaluation was performed (51, 52). Histograms of the bootstrap population mean PK parameter and variance estimates were also generated to assess the general shape of the distribution for each term in the model. The purpose of this was to further assess the precision of the final population PK parameter estimates in response to perturbations in the data and to assist with assessing potential areas of weaknesses and identifying limitations of the model.

### Calculation of secondary PK parameters and exposure estimates.

The individual *post hoc* PK parameter estimates (CL, *V_c_*, CL*_d_*_1_, CL*_d_*_2_, *V_p_*_1_, and *V_p_*_2_) were obtained from the final model and directly reported. These parameters were used to calculate secondary PK parameters, such as *V*_ss_ (which is equal to *V_c_* + *V_p_*_1_ + *V_p_*_2_), as well as the α-, β-, and γ-phase half-lives (*t*_1/2,α_, *t*_1/2,β_, and *t*_1/2,γ_, respectively). For most of the comparisons in this analysis, the individual *post hoc* PK parameters were also used to simulate predicted plasma plazomicin concentration-time data to generate plasma exposure estimates, such as *C*_max_, *C*_min_, and AUC_0–24_.

## Supplementary Material

Supplemental file 1
