# Supplementary material for: Population Pharmacokinetic Analyses for Plazomicin Using Pooled Data from Phase 1, 2, and 3 Clinical Studies
Source: Antimicrob Agents Chemother. 2019 Mar 27;63(4):e02329-18. doi: 10.1128/AAC.02329-18 (PMC6496156; doi:10.1128/AAC.02329-18)
Supplement: Supplemental file 1 [file AAC.02329-18-s0001.pdf]

SUPPLEMENTAL MATERIAL

FIG S1 Goodness-of-fit plots for the final population PK model for plazomicin. iPred, individual prediction; Pred, population prediction; OBS, observation; r^2, coefficient of determination.

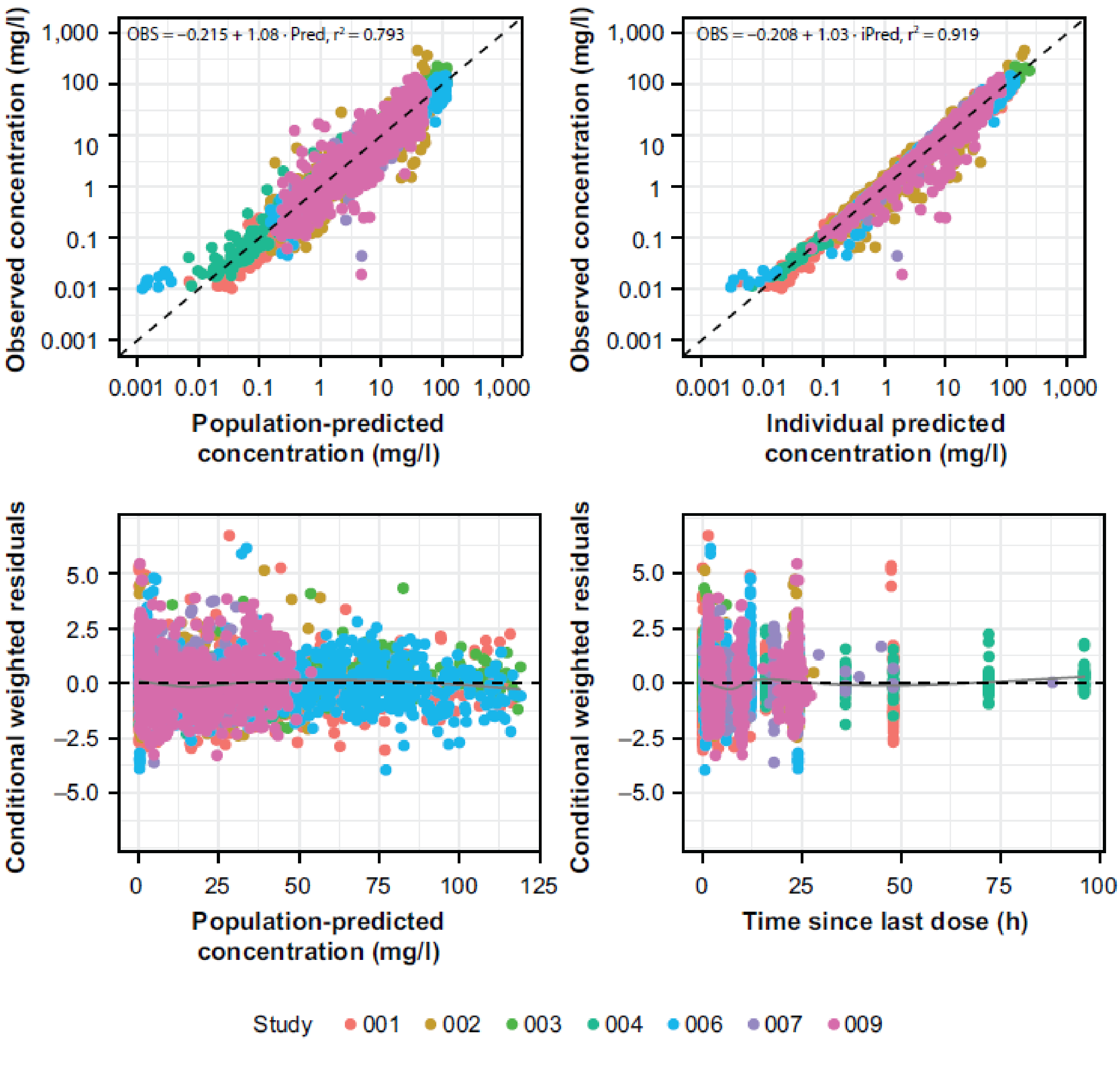

TABLE S1 Description of plazomicin data included in population PK analysis

Study	Subjects[a]/ samples at start	Outlier samples excluded	BLQ samples excluded[b]	Subjects/ samples at end	% of subjects/ samples retained for analysis
Phase 1					
001 (1)	28/1,354	1	0	28/1,353	100/99.9
003 (2)	30/350	0	0	30/350	100/100
004 (3)	24/313	1	0	24/312	100/99.7
006 (4)	61/941	3	36	61/902	100/95.9
Phase 2					
002 (5)	96/579	69	7	92/503	95.8/86.9
Phase 3					
007 (6)	48/462	0	0	48/462	100/100
009 (7)	286/1,143	32	3	281/1,108	98.3/96.9
Sum	573/5,142	106	46	564/4,990	98.4/97.0

[a]Number of subjects or patients included in the PK population of each study; [b]Plasma BLQ samples and outlier observations were retained in the dataset but flagged so that they were ignored by NONMEM.

BLQ, below the lower limit of quantification.

TABLE S2 Results of forward selection and backward elimination steps of covariate analysis to identify significant covariate relationships

Round	Parameter	Covariate[a]	MVOF	Change from comparator[a] (*df*)
Forward selection				
Base model			15,235	NA
1	Vc	Infection type	15,151	–84.18 (4)
2	Vp1	BSA	15,067	–83.87 (1)
3	CLd1	Age	15,003	–63.93 (1)
4	CL	Body weight	14,969	–33.80 (1)
5	Vc	Age	14,940	–29.57 (1)
6	CL	Infection type	14,910	–29.68 (4)
7	Vp1	Vasopressor use	14,889	–21.00 (1)
8	CLd2	Height	14,876	–12.70 (1)
9	CLd1	Infection type	14,849	–26.89 (4)
10	Vp1	Infection type	14,826	–23.39 (4)
11	Vp1	Age	14,811	–15.20 (1)
12	Vc	BSA	14,798	–12.98 (1)
13	Vp2	Body weight	14,783	–14.47 (1)
14	Vp2	Infection type	14,768	–15.74 (4)
15	CLd2	Infection type	14,750	–17.19 (4)

Round	Parameter	Covariate[a]	MVOF	Change from comparator[a] (*df*)
16	Vp2	Vasopressor use	14,740	–10.35 (1)
17	CL	Age	14,731	–10.09 (1)
18	No significant relationships identified			
Backward elimination				
Full multivariable model			13,755	NA
1	Vp2	Infection type	13,759	+4.38 (4)
2	Vc	Age	13,761	+1.96 (1)
3	CLd1	Age	13,763	+1.97 (1)
4	CL	Age	13,774	+10.2 (1)

[a]In order to be retained in the model, addition of a parameter–covariate relationship with one degree of freedom must have resulted in a 6.635-unit decrease in the MVOF in forward selection; removal of a parameter–covariate relationship with one degree of freedom must have resulted in a 10.83-unit increase in the MVOF in backward elimination.

Development of the full multivariable model was conducted between forward selection and backward elimination steps of covariate analysis.

After the backward elimination, refinement of the final covariate model was conducted to make the model more parsimonious. The assessments by proportional shifts in PK

parameters for infection types resulted in that the parameter–covariate relationships CL:cUTI and CL:HABP/VABP were dropped and CLd1:AP and CLd1:cUTI, Vp1:HABP/VABP and Vp1:BSI, Vc:AP and Vc:cUTI, Vc:HABP/VABP and Vc:BSI, Vp1:AP and Vp1:cUTI, CLd2:HABP/VABP and CLd2:BSI, and CLd2:AP and CLd2:cUTI were combined. The following bootstrap procedure resulted in the relationships of Vp1:vasopressor use, CLd1:HABP/VABP, CLd1:BSI, Vp1:HABP/VABP, and Vp1:BSI dropped off.

df, degrees of freedom; MVOF, minimum value of the o

References

1. Cass RT, Brooks CD, Havrilla NA, Tack KJ, Borin MT, Young D, Bruss JB. 2011. Pharmacokinetics and safety of single and multiple doses of ACHN-490 injection administered intravenously in healthy subjects. Antimicrob Agents Chemother 55:5874-5880.
2. Cass R, Kostrub CF, Gotfried M, Rodvold K, Tack KJ, Bruss J. 2013. A double-blind, randomized, placebo-controlled study to assess the safety, tolerability, plasma pharmacokinetics and lung penetration of intravenous plazomicin in healthy subjects, Poster 1637. European Congress of Clinical Microbiology and Infectious Diseases, April 27-30, 2013, Berlin, Germany.
3. Komirenko AS, Riddle V, Gibbons JA, Van Wart S, Seroogy JD. 2018. A Phase 1 Study To Assess the Pharmacokinetics of Intravenous Plazomicin in Adult

Subjects with Varying Degrees of Renal Function. Antimicrob Agents Chemother 62. ACCEPTED [need DOI to epub].

4. Gall J, Choi T, Riddle V, Van Wart S, Gibbons JA, Seroogy J. 2019. A phase 1 study of intravenous plazomicin in healthy adults to assess potential effects on the QT/QTc interval, safety, and pharmacokinetics Clin Pharmacol Drug Dev (in press).

5. Connolly LE, Riddle V, Cebrik D, Armstrong ES, Miller LG. 2018. A multicenter, randomized, double-blind, phase 2 study of the efficacy and safety of plazomicin compared with levofloxacin in the treatment of complicated urinary tract infection and acute pyelonephritis. Antimicrob Agents Chemother 62. doi: 10.1128/AAC.01989-17.

6. Connolly LE, Jubb A, O'Keefe B, Serio AW, Gall J, Riddle V, Krause KM, McKinnell JA, Zakynthinos E, Daikos G. 2017. Plazomicin (PLZ) associated with improved survival and safety compared to colistin (CST) in serious carbapenem-resistant Enterobacteriaceae (CRE) infections: Results of the CARE study, Abstract 5831. American Society for Microbiology Microbe, June 1-5, 2017, New Orleans, LA.

7. Cloutier DC, Miller LG, Komirenko A, Cebrik D, Keepers T, Krause KM, Connolly LE, Wagenlehner FME. 2017. Evaluating once-daily plazomicin versus meropenem for the treatment of complicated urinary tract infection (cUTI) and acute pyelonephritis (AP): Results from a phase 3 study (EPIC), Oral presentation. American Society for Microbiology Microbe, June 1-5, 2017, New Orleans, LA.
